# Genetic Dissection of Seed Dormancy using Chromosome Segment Substitution Lines in Rice (*Oryza sativa* L.)

**DOI:** 10.3390/ijms21041344

**Published:** 2020-02-17

**Authors:** Shaowen Yuan, Yuntong Wang, Chaopu Zhang, Hanzi He, Sibin Yu

**Affiliations:** 1 College of Plant Science and Technology, Huazhong Agricultural University, Wuhan 430070, Hubei, China; wenshaoY@webmail.hzau.edu.cn (S.Y.); zchaopu@163.com (C.Z.); 2 National Key Laboratory of Crop Genetic Improvement, Huazhong Agricultural University, Wuhan 430070, Hubei, China; wangyt@biomarker.com.cn; 3 Biomarker Technologies Corporation, Beijing 101300, China

**Keywords:** seed dormancy, quantitative trait locus, ABA, seed germination, chromosome segment substitution lines, linkage mapping

## Abstract

Timing of germination determines whether a new plant life cycle can be initiated; therefore, appropriate dormancy and rapid germination under diverse environmental conditions are the most important features for a seed. However, the genetic architecture of seed dormancy and germination behavior remains largely elusive. In the present study, a linkage analysis for seed dormancy and germination behavior was conducted using a set of 146 chromosome segment substitution lines (CSSLs), of which each carries a single or a few chromosomal segments of Nipponbare (NIP) in the background of Zhenshan 97 (ZS97). A total of 36 quantitative trait loci (QTLs) for six germination parameters were identified. Among them, *qDOM3.1* was validated as a major QTL for seed dormancy in a segregation population derived from the *qDOM3.1* near-isogenic line, and further delimited into a genomic region of 90 kb on chromosome 3. Based on genetic analysis and gene expression profiles, the candidate genes were restricted to eight genes, of which four were responsive to the addition of abscisic acid (ABA). Among them, LOC_Os03g01540 was involved in the ABA signaling pathway to regulate seed dormancy. The results will facilitate cloning the major QTLs and understanding the genetic architecture for seed dormancy and germination in rice and other crops.

## 1. Introduction

Seed dormancy is an important evolutionary trait. It can optimize the distribution and timing of germination over time in nature [1]. Seed dormancy also plays an important role in agricultural production. Extremely strong dormancy leads to a low germination rate in the field, the irregular emergence of seedlings and an impact on sowing time, and can even affect the final yield. On the contrary, too weak dormancy leads to pre-harvest sprouting (PHS), especially in the high temperature and rainy environment during seed maturity. The economic loss caused by pre-harvest sprouting has become an important factor restricting the yield of cereal crops (such as rice, wheat, maize, etc.) [2], and seriously affects the sowing quality and processing quality of crops, and even causes the change of storage quality [3]. Therefore, crop seeds require a well-balanced level of dormancy to ensure a high rate of germination and to control pre-harvest sprouting in the field.

Seed dormancy is a highly complex trait and largely influenced by genetic and environmental factors [4]. Recent progress in plant genomics and various genetic populations has facilitated the identification of quantitative trait loci (QTLs) for seed dormancy in many species, for example, in *Arabidopsis* [5], *Lepidium sativum* [6], oilseed rape [7], sorghum [8], barley [9], and wheat [10,11,12].

By using different genetic populations constructed from cultivated rice, wild rice, and weedy rice, more than 160 QTLs have been identified that affect the germination or dormancy of rice (available online: https://archive.gramene.org/qtl/). For example, five seed dormancy QTLs were detected by BC1 and F_2_ populations constructed from rice variety N22 with strong dormancy and two weak dormant varieties [13]. Three seed dormancy QTLs were mapped using the chromosomal segment substitution line (CSSL) population and their derived F_2_ populations constructed by the strong dormant variety Nona Bokra and the weak dormant variety Koshihikari [14]. By using a population of recombinant inbred lines (RILs), nine seed dormancy QTLs were identified in three developmental stages [15]. In addition, besides biparental genetic populations, genome-wide association analysis across natural accession also revealed genetic variation of seed dormancy among rice natural populations, and the analysis can be used to identify new candidate genes related to seed dormancy [16,17].

In 2010, Japanese scientists isolated the first rice seed dormancy gene *Seed dormancy 4* (*Sdr4*) by map-based cloning [18]. *Sdr4* can be positively regulated by the seed maturation-related gene *OsVP1* and two *Arabidopsis* dormancy gene *Delay of Germination 1 (DOG1)* [19] homologous genes, thereby enhancing seed dormancy. After that, several rice seed dormancy genes were identified and proved to be involved in the hormonal regulation of seed dormancy. By rice mutant screening, *PHS8* was isolated to be a starch debranching enzyme named isoamylase1, and it determined seed dormancy and germination by affecting abscisic acid (ABA) signaling [20]. The rice *GERMINATION DEFECTIVE 1* regulated seed germination by integrating gibberellin acid (GA) and carbohydrate metabolism [21]. A weedy red rice dormancy QTL (*SD7-1/Rc*) was identified as a basic helix-loop-helix transcription factor that controls ABA synthesis, influencing red pericarp color and seed dormancy [22]. By map-based cloning, a gibberellin synthesis gene *OsGA20ox2* was identified within QTL *Seed Dormancy1-2* (*qSD1-2*) [23]. *OsGA20ox2* (the green revolutionary gene *SD1*) is involved in the biosynthesis of GA, regulating the development of endosperm-imposed dormancy in rice.

Hormonal regulation may be a highly conserved mechanism of seed dormancy among many species. The balance of ABA and GA or other hormones plays crucial roles in the regulation of seed dormancy and germination [24,25]. ABA is an essential positive regulator of both dormancy induction during seed maturation and maintenance of the dormant state after imbibition [26,27,28]. In *Arabidopsis*, *DOG1* was the first cloned dormancy QTL, and encoded a protein with unknown functional domain [19] and had conserved function throughout many species. In recent years, *DOG*1 was reported to play a regulatory role in ABA signaling. It encodes for a plant-specific protein that enhances ABA signaling through its binding to protein phosphatase 2C (PP2C) ABA HYPERSENSITIVE GERMINATION1 (AHG1) and AHG3 [29,30]. In addition, *DOG1* may mediate a conserved seed coat dormancy mechanism in the temperature- and GA-dependent pathways [31]. Besides *DOG1*, the previously mentioned seed dormancy genes, such as *PHS8* and *SD7-1/Rc*, were involved in either ABA metabolism or signaling pathway. Thus, it is worthwhile to investigate whether there are more genes involved in the hormonal regulation of seed dormancy.

Here, we presented the identification of QTLs for seed dormancy in a set of genome-wide single nucleotide polymorphism (SNP) genotyped chromosomal segment substitution lines (CSSLs) by backcrossing and marker-assisted selection, in which *japonica* Nipponbare (NIP) was the donor parent and the recurrent parent was *indica* Zhenshan 97 (ZS97). The CSSL population, which comprised 146 lines, was developed and genotyped in a previous study [32,33] and has not been used for a seed dormancy study.

Therefore, the objectives of the present study were to dissect the genetic base of seed dormancy and germination performances in the CSSL population, and to fine map the major QTLs using the CSSL-derived population. Moreover, we investigated how the candidate gene was involved in the ABA regulation of seed dormancy.

## 2. Results

### 2.1. Seed Dormancy Variation in Parents

**Figure 1:**
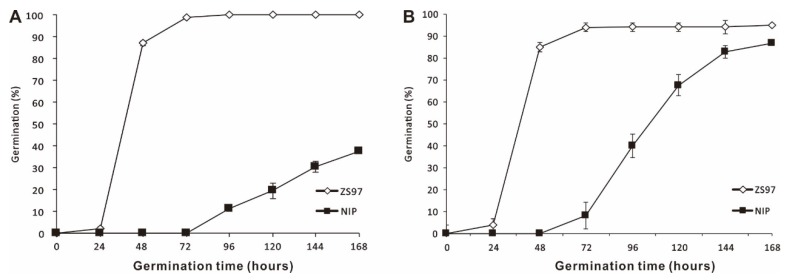
(**A**) Germination behavior of freshly harvested *japonica* variety Nipponbare (NIP) and *indica* variety Zhenshan97 (ZS97) seeds; (**B**) germination behavior of after-ripened NIP and ZS97 seeds.

The germination assay for the freshly harvested seed of the parental lines, which were *indica* variety Zhenshan97 (ZS97) and *japonica* variety Nipponbare (NIP), was performed. The number of germinated seeds was counted daily for seven consecutive days and the germination percentage was calculated each day to construct cumulative germination curves. ZS97 and NIP exhibited significant difference in seed dormancy, in which ZS97 had significantly higher germination percentage (100%) than NIP (37%) at seven days (168 h of germination) (Figure 1A). Dry seeds were treated at 43 °C for three days to break seed dormancy (which was called after-ripened seeds) and then underwent a seed germination experiment. After seed dormancy was broken, the germination percentage of seven days (168 h) of the parental lines was almost the same (around 90%) (Figure 1B). However, the germination rate of NIP was still lower as compared to ZS97. At two days of germination (48 h), the radicle protrusion of ZS97 was 86%, whereas no radicle protrusion occurred for NIP (Figure 1B).

### 2.2. Seed Dormancy Variation in the CSSL Population

The CSSL population, which comprised 146 lines, was developed and genotyped in a previous study [32,33] and the lines were used to dissect the genetic architecture of seed dormancy underlying this population.

Seed germination was analyzed using the six germination parameters from the Germinator package [34], which were G_max_ (maximum germination percentage at seven days); G_3d_ (germination percentage atthree3 days), T_50_ (germination speed: time to reach 50% germination of the total number of germinated seeds), U_8416_ (germination uniformity: time interval between 16% and 84% of viable seed to germinate), AUC (area under the curve), and GI (germination index). The mean performance of the CSSL population is presented in Table 1. The frequency distribution for the six germination parameters of the CSSL population showed large variation (Figure S1, Supplementary Materials). These results indicated that there might be seed dormancy QTLs in this CSSL population.

### 2.3. Seed Dormancy QTL Detection in CSSL Population

**Figure 2:**
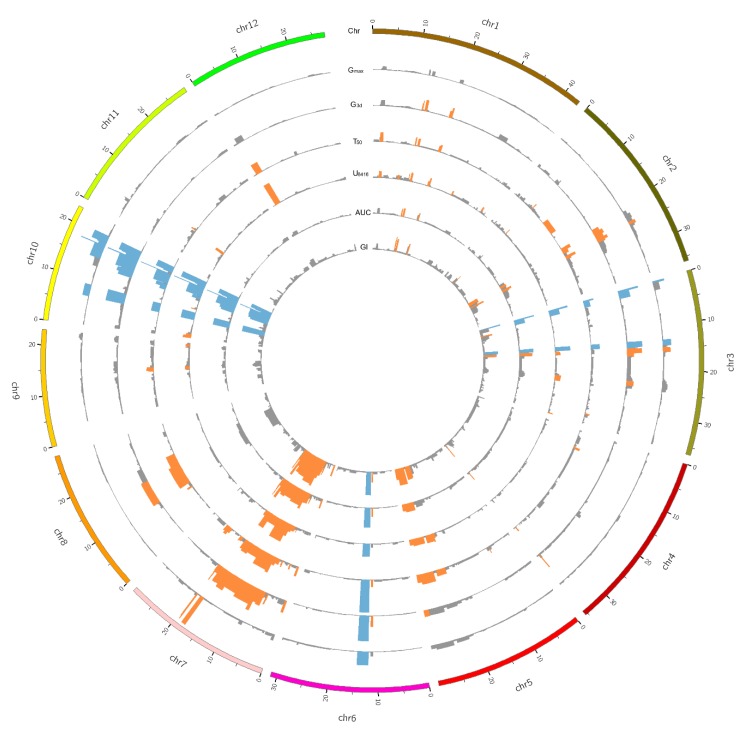
Circos plot illustrating the quantitative trait loci (QTLs) of six germination parameters. Chr: Size of the 12 chromosomes of *Oryza sativa*; G_max_, maximum germination percentage of seven days germination; G_3d_, germination percentage at three days; T_50_, time to reach 50% germination of the total number of germinated seeds; U_8416_, germination uniformity, which is time interval between 84% and 16% of viable seed to germinate; AUC; area under the germination curve until 168 h; GI, germination index. Blue indicates significant QTLs identified by all six germination parameters. Orange indicates significant QTLs identified in the corresponding germination parameter. Gray indicates non-significant QTLs.

To determine the genetic regions controlling seed dormancy, the CSSL population was genotyped by the RICE6K SNP array. A total of 518 bins (defined as Bin1 to Bin518) across the whole genome were obtained [33]. QTL mapping was carried out by using ridge regression analysis with the 518 bins, and it identified 9, 19, 25, 23, 17, and 21 QTLs for G_max_, G_3d_, T_50_, U_8416_, AUC, and GI, respectively (Figure 2). Detailed information about the *p*-value, phenotypic variation explained, and the effect of the identified QTLs are shown in Table 2.

In total, 36 QTLs were detected for seed dormancy using the six parameters in the CSSL population (Table 2). Among the six parameters, U_8416_ explained the highest phenotypic variance (77.9%) with 23 QTLs detected, while T_50_ explained 71.5% of phenotypic variance with the largest number of QTLs detected (25 QTLs). G_3d_, AUC, and GI explained 77%, 66.3%, and 52.7% of phenotypic variance with 19, 17, and 21 QTLs detected, respectively. G_max_ only detected 9 QTLs and explained 50.5% of phenotypic variance. The 36 QTLs were distributed on each of the chromosome in which both chromosome 6 and 10 had the highest -log10(*p*) value (15.7) for U_8416_, and the phenotypic variance were 6.5% and 10.4%, respectively. Among all 36 QTLs, 10 QTLs were identified or cloned previously for seed dormancy, suggesting the consistency of our QTL analysis with others. The other remaining 26 QTLs may be new ones for seed dormancy, as they do not contain any QTLs for dormancy in rice that have been described before (https://archive.gramene.org/qtl/).

Five out of thirty-six QTLs were common QTLs detected in all six parameters and distributed on chromosomes 3, 6, and 10. One common QTL (*qDOM3.3*) on chromosome 3 was identified as a seed dormancy QTL in the “Asominori×IR24” CSSL population [35], and *qDOM10.3*, which was detected in all six parameters, contained a gene, namely *OsFbx352* [36], that plays a regulatory role in the regulation of glucose-induced suppression of seed germination by targeting ABA metabolism. The other three common QTLs (*qDOM3.1*, *qDOM6.2*, and *qDOM10.2*) have not been reported before. *qDOM3.1* was detected in almost the same region on the upper end of chromosome 3 by the six germination parameters, suggesting the robustness of this QTL in the present CSSL population. U8416 of *qDOM3.1* had the highest -log10(P) value (9.2) among the six parameters and explained 8.3% of phenotypic variance.

### 2.4. Verification of qDOM3.1 for Seed Dormancy

To validate and fine map *qDOM3.1*, one line, namely NQ96, in the CSSL population was selected (Figure S2, Supplementary Materials). It carries a NIP substitution segment encompassing *qDOM3.1* on top of chromosome 3 in the ZS97 genetic background, with another NIP substitution segment on chromosome 9. The germination behavior of NQ96 was significantly lower and slower than ZS97 (Table 3). This indicated that the introduced NIP segment contained the QTL of seed dormancy. To confirm the genetic effect of the *qDOM3.1* on seed dormancy, we generated an F_2_ segregating population comprising 338 individuals by crossing NQ96 with ZS97. The F_2_ population was genotyped using ten polymorphic markers in the *qDOM3.1* region and one polymorphic marker on the other introgressed segment on chromosome 9. There was no significant difference on seed dormancy for NIP and ZS97 allele on chromosome 9 with marker RM410, denoting that the introgressed segment on chromosome 9 had no effect on seed dormancy. Thus, NQ96 only contained *qDOM3.1*, which had a genetic effect on seed dormancy.

**Figure 3:**
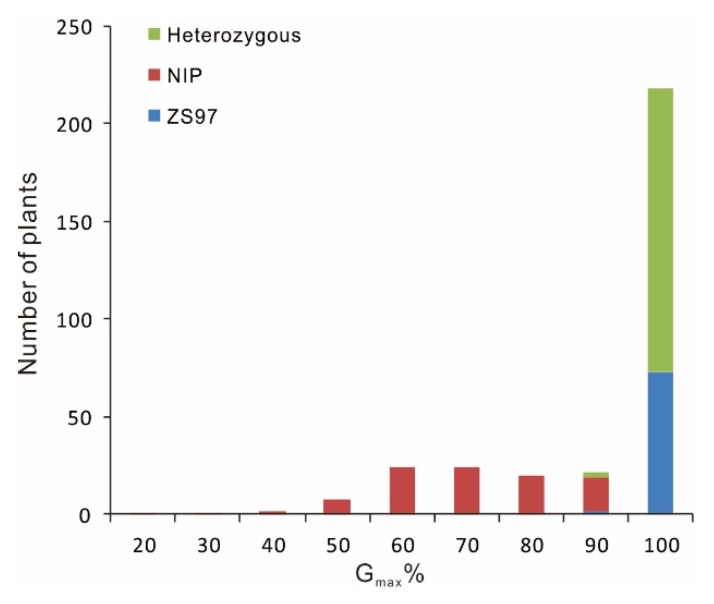
Frequency distribution of G_max_ in F_2_ segregation population.

To determine the genetic effect of *qDOM3.1*, we performed a genetic segregation analysis of seed dormancy using the *qDOM3.1*-derived F_2_ population. Frequency distribution of G_max_ in the population indicated that *qDOM3.1* from ZS97 was dominant (Figure 3).

**Figure 4:**
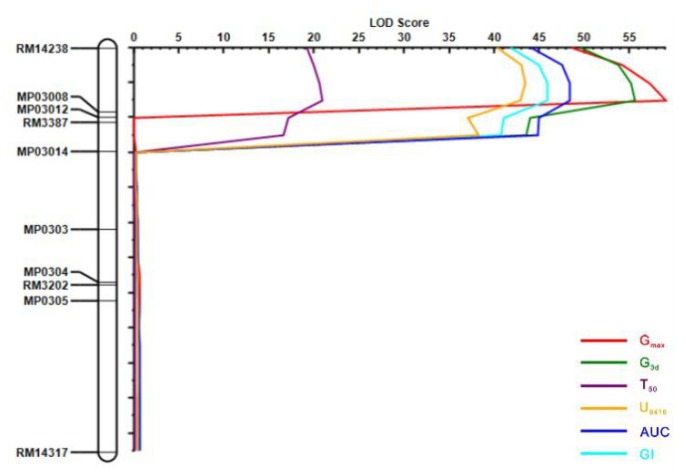
Verification of the QTL effect in the F_2_ primary segregation population. QTL scans along chromosome 3 for the six indexes in the CSSL-derived F_2_ population from the cross of NQ96 and ZS97. Logarithm of odds profile of QTL region on chromosome 3 in the F_2_ population, showing a QTL (*qDOM3.1*) for G_max_, G_3d_, T_50_, U_8416_, AUC, and GI.

Afterward, based on the genotyping results of ten polymorphic markers distributed within the target region RM14238-RM14317 for the F_2_ individuals, *qDOM3.1* was detected in the interval RM14238-MP030012 (approximately 252 kb) with a logarithm of the odds (LOD) score peaked around MP03008 (Figure 4), which explained 69.9%, 75.4%, 73.2%, 71.4%, 38.3%, and 46.5% of the phenotypic variance in G_max_, G_3d_, AUC, GI, U8416, and T50, respectively.

### 2.5. Fine-Mapping of qDOM3.1 for Seed Dormancy

**Figure 5:**
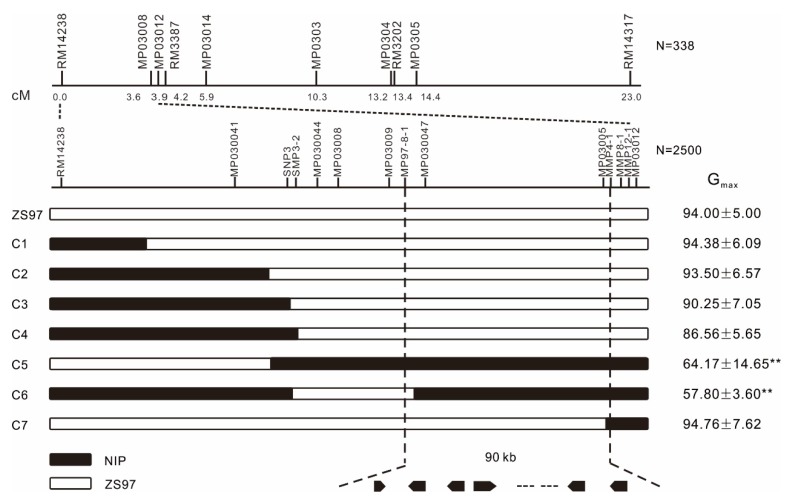
Fine mapping of *qDOM3.1*. The QTLs narrowed down to the region flanked by MP97-8-1 and MMP4-1 on the upper end of chromosome 3. Some important recombinant plants derived from a large F_2_ group generated by selfing a single individual heterozygous at the *qDOM3.1* region and divided into 7 groups based on their genotypes. G_max_ (mean ± SD) (%) is given on the right for each genotype. The phenotypes of each recombinant individual were evaluated by germination experiments. ** Indicates significant difference at *p* < 0.01 by Dunnett’s test against the control.

To further fine map *qDOM3.1*, we selected the heterozygous lines in the CSSL-derived F_2_ population flanked by the markers RM14238 and MP030012, and self-pollinated these heterozygous lines to generate a larger segregating population (*n* = 2500). Through genotyping with 14 additional markers, seven informative recombinants with *qDOM3.1* were identified. A progeny test of the informative recombination plants delimited *qDOM3.1* for seed dormancy to a 90 kb region (Figure 5 and Figure S3, Supplementary Materials). This region encompassed 19 open reading frames (ORFs) according to the RGAP database (available online: http://rice.plantbiology.msu.edu/, Release 7).

**Figure 6:**
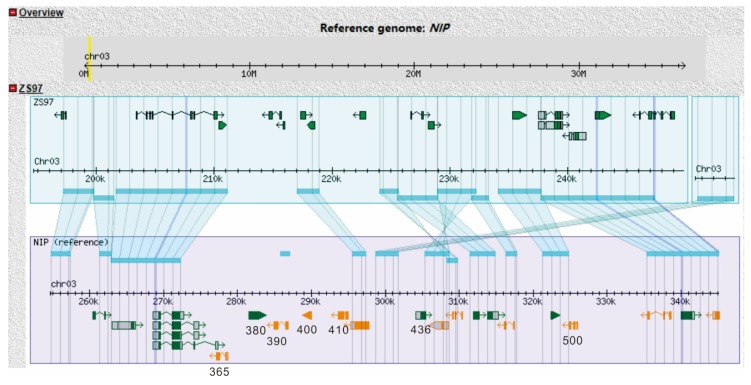
Chromosomal synteny analysis of ZS97 and NIP of candidate region on chromosome 3. The genes in which ZS97 was not contained are indicated with the final three numbers of the gene ID (e.g., LOC_Os03g01365 is displayed as 365). In NIP panel, green gene symbol means gene direction from left to the right and orange gene symbol means gene direction from right to the left.

The 19 genes included 11 expressed proteins, 1 transposon protein, 2 retrotransposon proteins, 1 hypothetical protein, and 4 genes with functional annotation. The chromosomal synteny analysis between NIP and ZS97 showed there were 7 genes missing in the ZS97 (available online: http://rice.hzau.edu.cn/cgi-bin/gb2/gbrowse_syn/3rice_syn/) (Figure 6). As the candidate gene should be dominant in ZS97 (Figure 3), those 7 genes were ruled out from the candidate genes. Thus, only 12 genes remained, including 10 expressed proteins, 1 gene annotated as tubulin/FtsZ domain-containing protein (LOC_Os03g01530), and another annotated as DNA-binding protein (LOC_Os03g01540). According to the expression profile in the database (http://rice.plantbiology.msu.edu/expression.shtml), 4 genes (LOC_Os03g01430, LOC_Os03g01450, LOC_Os03g01460, and LOC_Os03g01520) had no expression or very low expression among all the tissues. Therefore, those 4 genes were unlikely to be our candidate genes, leaving 8 genes as candidate genes. The expression profiles were obtained from the RiceXPro website (available online: http://ricexpro.dna.affrc.go.jp/) (Figure S4, Supplementary Materials). None of the 8 genes were seed-specific expressed, except LOC_Os03g01360 that had a relatively higher expression in embryo from 7 days after flowering until 42 days after flowering. Based on the sequence comparison between NIP and ZS97, only LOC_Os03g01530 had no amino acid change in the coding region; all the other 7 genes contained at least one missense variant.

### 2.6. qDOM3.1 Increased Seed Endogenous ABA Content and ABA Sensitivity

**Figure 7:**
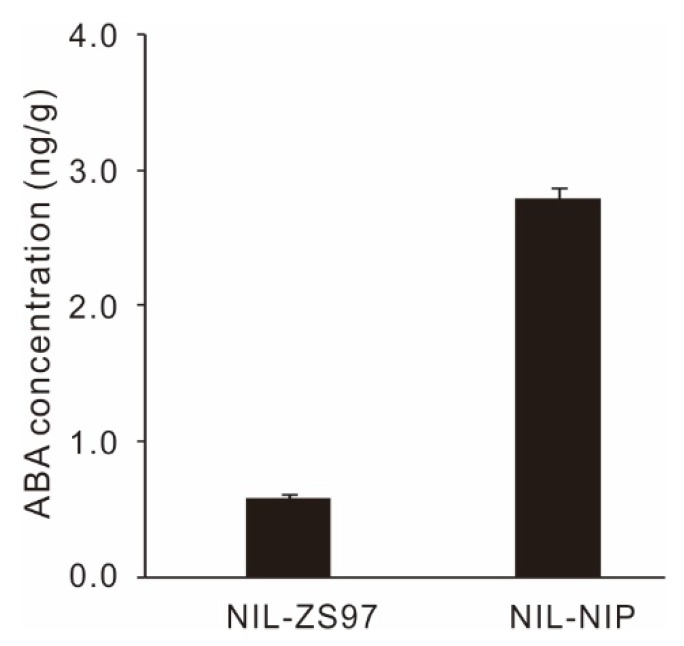
Endogenous ABA concentration in near-isogenic line (NIL)-ZS97 and NIL-NIP.

ABA plays an essential role in the regulation of seed dormancy [4,24,37]. The endogenous ABA level was measured in the near-isogenic line (NIL) of *qDOM3.1* (NIL-NIP) and the corresponding background line (NIL-ZS97). NIL-NIP had an ABA level almost five times higher than that of the NIL-ZS97 (Figure 7).

**Figure 8:**
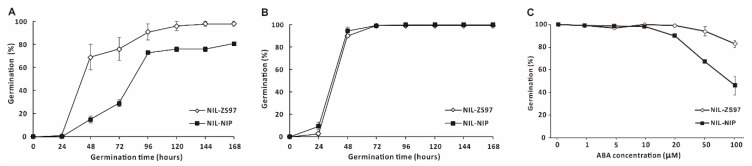
(**A**) Germination behavior of freshly harvested NIL-ZS97 and NIL-NIP seeds; (**B**) germination behavior of after-ripened NIL-ZS97 and NIL-NIP seeds; (**C**) ABA sensitivity of NIL-ZS97 and NIL-NIP.

Subsequently, the ABA sensitivity was investigated in the near-isogenic lines (NIL-NIP and NIL-ZS97) for freshly harvested seeds (Figure 8A) and after-ripened seeds (Figure 8B). The germination percentage and germination speed were significantly lower and slower in NIL-NIP compared with NIL-ZS97 (Figure 8A). Freshly harvested seeds were treated in 43 °C for three days to break seed dormancy, and the germination behavior was almost the same for after-ripened NIL-NIP and NIL-ZS97 (Figure 8B). Then, the pair of near-isogenic lines (after-ripened) were treated in a series of ABA solution to investigate ABA sensitivity. Up to 10μM ABA had no significant effect on seed germination, whereas 20–100μM ABA significantly decreased seed germination of NIL-NIP compared with NIL-ZS97 (Figure 8C). Thus, *qDOM3.1* was sensitive to ABA treatment. Therefore, we hypothesized that the target region may contain an ABA responsive gene.

### 2.7. Candidate Gene Expression Changes Upon ABA Treatment

**Figure 9:**
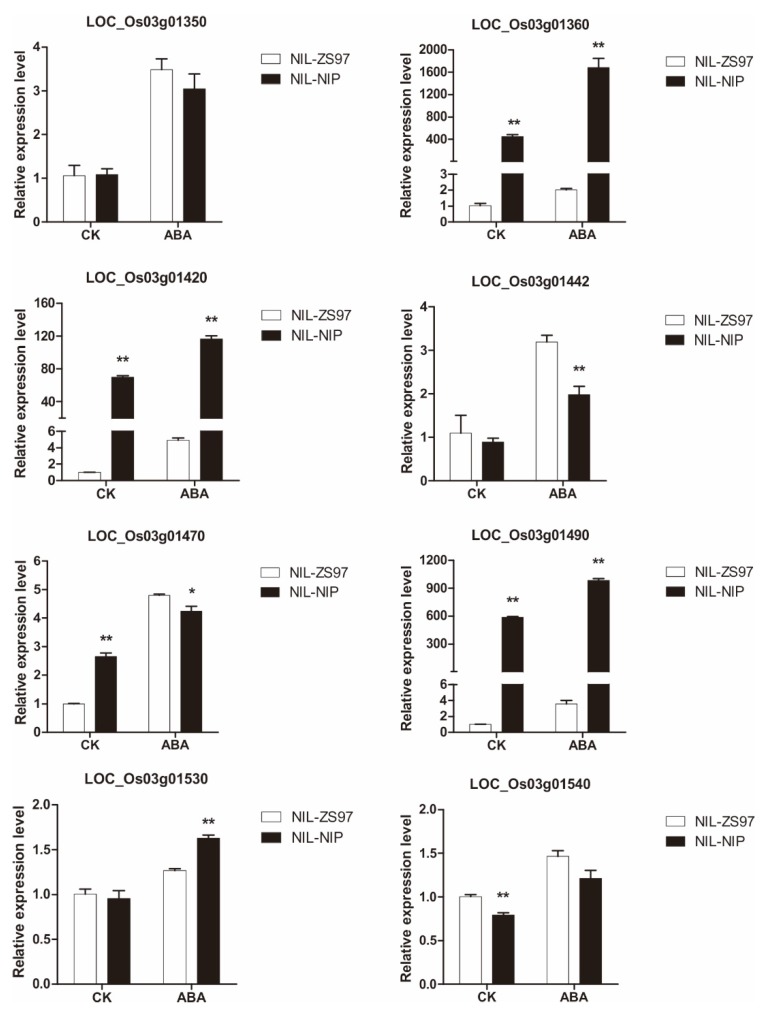
Relative mRNA abundance level of eight candidate genes under *qDOM3.1* using after-ripened NIL-ZS97 and NIL-NIP before (CK) and after 20 μM of abscisic acid (ABA) treatment (ABA). * and ** indicate significant differences at *p* < 0.05 and *p* < 0.01 using the Student’s *t*-test of NIL-NIP against NIL-ZS97, respectively. CK of NIL-ZS97 in each figure was determined as 1.

For that reason, the gene expression of eight candidates was measured upon ABA treatment (20 μM), using after-ripened NIL-NIP and NIL-ZS97. In total, four out of eight candidate genes were ABA responsive genes (Figure 9). LOC_Os03g01442’s expression level had no significant difference in non-treated NIL-NIP and NIL-ZS97 (CK); however, upon ABA treatment, the expression level was significantly higher in NIL-NIP than NIL-ZS97. LOC_Os03g01540 had the opposite effect, which was lower in non-treated NIL-NIP than in NIL-ZS97 and, upon ABA treatment, the expression level had no difference in NIL-NIP and NIL-ZS97. LOC_Os03g01530’s expression level was significantly increased upon ABA treatment. LOC_Os03g01470’s expression level was significantly higher in non-treated NIL-NIP than NIL-ZS97, and, after ABA treatment, the expression level was significantly lower in NIL-NIP compared with NIL-ZS97. The other four candidate genes had the same trend before and after the ABA treatment.

Therefore, if the expression of the candidate gene was changed by the addition of ABA, they were unlikely to be our candidate genes. Therefore, there were four candidate genes under *qDOM3.1*, including two expressed proteins (LOC_Os03g01442 and LOC_Os03g01470), one tubulin/FtsZ domain-containing protein (LOC_Os03g01530), and one DNA-binding protein (LOC_Os03g01540). However, we cannot rule out the posttranslational modifications of the candidate gene, such as phosphorylation/dephosphorylation. More experimental evidence is needed.

## 3. Discussion

### 3.1. Seed Dormancy QTL Analysis

Seed dormancy in rice is generally a complex trait and is controlled by multiple genes. In our study, the genetic architecture of seed dormancy was examined in the NIP/ZS97 CSSL population with each line carrying one or a few different introgressed segments from NIP and otherwise sharing the uniform genetic background of ZS97. Each introgression segment was defined by high-density SNP markers. The CSSL population has several advantages over other mapping populations such as F_2_, BC1, and RIL [38,39,40]. First, the detection power of QTLs in CSSLs was higher than that of other mapping populations reported. In total, 36 QTLs for seed dormancy were identified in this CSSL population using six germination parameters (Table 2) and the six germination parameters explained from 50.5% to 77.9% of phenotypic variance. However, only four seed dormancy QTLs were detected in a double haploid (DH) population [41]. Four [42] and nine [15] seed dormancy QTLs were identified by two different RIL populations, respectively. Second, it is easier to develop a secondary F_2_ population derived from a cross between a CSSL line containing the target QTL and the recurrent parent for fine mapping [38,43]. In our study, by developing an F_2_ segregation population, one of the novel seed dormancy QTLs, namely *qDOM3.1*, was delimited to 90 kb (Figure 5).

Among all 36 QTLs, 10 were detected or cloned previously for seed dormancy (Table 2). The results implied that our NIP/ZS97 population and QTL analysis method turned out to be efficient to detect seed dormancy QTLs across the whole genome. The first cloned seed dormancy gene in rice *Sdr4* was only detected in T_50_ (*qDOM7.5*), implying that our population had mild seed dormancy level. *qDOM7.4* covered the gene *SD7-1/Rc* and was detected in all six parameters except G_max_. *SD7-1/Rc* was a pleiotropic gene that most likely controlled the dormancy and pigment traits by regulating ABA and flavonoid biosynthetic pathways, respectively [22]. *OsFbx352*, which was located under *qDOM10.3*, was detected in all six parameters. It was involved in the regulation of glucose-induced suppression of seed germination by targeting ABA metabolism [36]. Besides the QTLs co-located with the previous study, there were 26 new QTLs identified in the present study; therefore, both the plant material and abundant QTL information will facilitate the use of the dormancy alleles in other breeding programs or other research studies.

### 3.2. Candidate Gene Analysis

In the present study, three new QTLs were detected in all six germination parameters, namely *qDOM3.1*, *qDOM6.2*, and *qDOM10.2*. Through a CSSL-derived F_2_ population, the major effect of *qDOM3.1* on seed dormancy was validated for the first time using the six seed dormancy-related parameters and delimited to a 90 kb region (Figure 4 and Figure 5 and Figure S3), which included 19 candidate genes. As the heterozygous line had the same phenotype as ZS97 (Figure 3), the candidate gene in ZS97 should be dominant. Based on the chromosomal synteny analysis and the gene expression profile in different tissues, we deducted that eight genes left as candidate genes. For the eight candidate genes, LOC_Os03g01540 was annotated as DNA-binding protein, LOC_Os03g01530 was annotated as tubulin/FtsZ domain-containing protein, and the other six candidate genes were annotated as expressed protein (based on the RGAP database: http://rice.plantbiology.msu.edu/, Release 7).

Hormonal regulation may be a highly conserved mechanism of seed dormancy among many species. ABA plays an essential role in the regulation of seed dormancy [4,24,37]. In the present study, we investigated the ABA content in freshly harvested NIL-NIP and NIL-ZS97, and found out the ABA content was almost five times higher in NIL-NIP than in NIL-ZS97 (Figure 7). Subsequently, the ABA sensitivity assay demonstrated that NIL-NIP was indeed more sensitive to high ABA solutions than NIL-ZS97 (Figure 8C). Based on those results, we assumed that our candidate gene could be responsive to ABA treatment, and the two alleles in NIP and ZS97 should respond differently. Thus, four candidate genes were selected (Figure 9), which were two expressed proteins (LOC_Os03g01442 and LOC_Os03g01470), one tubulin/FtsZ domain-containing protein (LOC_Os03g01530), and one DNA-binding protein (LOC_Os03g01540).

The DNA-binding protein (LOC_Os03g01540) had 60% of protein sequence similarity with AT-hook motif DNA-binding family protein in *Arabidopsis* and 56.35% of similarity with AT-hook protein 1 in *Oryza sativa*. A recent study showed that the DNA-binding protein AT-Hook-Like 10 (AHL10) could be dephosphorylated by a protein phosphatase Highly ABA-Induced1 (HAI1), which was involved in abiotic stress and abscisic acid signaling. AHL10 phosphorylation was crucial for hormone-related genes during drought stress [44]. Therefore, we thought our candidate gene LOC_Os03g01540 was somehow involved in the ABA signaling pathway to regulate seed dormancy. However, further research will be needed to prove our hypothesis.

LOC_Os03g01530 is one of the isotype genes controlling β-tubulin, which is a basic component of microtubules. Proteomic analysis of rice embryo showed that LOC_Os03g01530 was upregulated during seed germination, suggesting its possible role in seed germination [45]. LOC_Os03g01490 was annotated as expressed protein. A research study showed that it is a functional new chimerical gene for *Oryza sativa* ssp. *japonica* by comparing *Oryza sativa* ssp. *japonica* and its five wild progenitors [46], although its function was not revealed. Another research paper identified LOC_Os03g01490 and LOC_Os03g01470 as phosphopeptides [47]. The other four candidate genes were annotated as expressed protein, and very limited information was available based on a literature search.

## 4. Materials and Methods

### 4.1. Plant Materials

#### 4.1.1. Experimental Design

The plant materials were planted at the experimental field of Huazhong Agricultural University at Wuhan (29.58°N, 113.41°E). The temperature during the late stage of maturity ranged from 25 °C to 30 °C, which was normal for rice growth and seed maturation. The first flowering date of each plant was recorded by the emergence of the first panicle from the leaf sheath [13].

Seeds were harvested from the individual plants at 35 days after flowering, which was defined as freshly harvested seeds and then equilibrated about 5–6 days at 15% relative humidity (called freshly harvested seeds), and then stored at −20 °C for subsequent analyses. A three-day heat treatment at 43 °C for dry seeds was used to break seed dormancy, and the seeds were called after-ripened seeds. All the analyses were performed with three biological replicates.

#### 4.1.2. CSSL Population

The CSSL population was developed and genotyped in a previous study [32,33]. The details are as follows. A set of chromosomal segment substitution lines (CSSLs) comprising 146 lines was developed in a previous study [32], in which the donor parent was *japonica* variety Nipponbare (NIP) and the recurrent parent *indica* was variety Zhenshan97 (ZS97). The CSSL population was genotyped previously, of which 518 bins were defined with a median size of 400 kb [33].

#### 4.1.3. CSSL Line-Derived Population

One of the CSSLs (NQ96), which contains the target QTL, was selected to backcross with ZS97 to generate an F_2_ population (called CSSL-derived population) for QTL validation. A total of 338 F_2_ plants and the F_2:3_ families of several recombinants were screened with polymorphic markers to identify the respective genotype.

#### 4.1.4. Plant Materials for ABA Content Measurement, ABA Sensitivity, and qRT-PCR Analysis

A pair of near-isogenic lines (NILs) containing the NIP alleles (NIL-NIP) and ZS97 alleles (NIL-ZS97), respectively, at the target QTL (*qDOM3.1*) in a common background of ZS97 were developed based on polymorphic markers. NIL-NIP and NIL-ZS97 were used for ABA content measurement, ABA sensitivity, and qRT-PCR analysis.

### 4.2. Seed Trait Measurement

In all, 50 seeds of each sample were spread on moistened filter paper in Petri dishes in a 25 °C growth chamber for germination experiments. The number of germinated seeds was counted daily for seven consecutive days to construct cumulative germination curves. Germination was defined as the length of the protruded radicle by 3–5 mm. Germination tests for the after-ripened seeds were also conducted as described in the above method.

Germination was scored using the Germinator package [34]. We calculated the six relevant parameters from the germination curve. The parameters included maximum germination percentage of seven days germination (G_max_); germination percentage at three days (G_3d_); germination speed, which is the time to reach 50% germination of the total number of germinated seeds (T_50_); germination uniformity, which is the time interval between 84% and 16% of viable seed to germinate (U_8416_) (16% and 84% stands for −1SD and +1SD, respectively); and area under the germination curve (AUC). Germination index (GI) was calculated by the method of Cao et al. [48]: (GI = Σ(Gt/Tt), where Gt is the number of the germinated seeds on Day t, and Tt is the time corresponding to Gt in days.

### 4.3. DNA Extraction and Marker Analysis

DNA was isolated from 2 cm long leaves using the cetyl trimethylammonium bromide (CTAB) method [49]. According to the sequence variation between NIP and ZS97 (available online: http://ricevarmap.ncpgr.cn/v2/), single nucleotide polymorphism (SNP) markers and insertion/deletion (Indel) markers in the target region were developed (Table S1, Supplementary Materials). The primers used for nucleotide variation analysis were designed according to the Nipponbare reference genome by Primer 3 (available online: http://redb.ncpgr.cn/modules/redbtools/primer3.php). Polymerase chain reaction (PCR) amplification and gel electrophoresis for marker genotype analysis were conducted following the methods described previously [50]. PCR products were sequenced by Sangon Biotech (Shanghai, China) and the sequences were analyzed using Sequencher 5.0 (Gene Codes Corporation, Ann Arbor, MI, USA).

### 4.4. QTL Analysis and Linkage Mapping

Germination percentage (x) such as G_max_ and G_3d_ was transformed by arcsine(x)0.5 to make the trait mean independent from the variance.

Based on the SNP genotypes, a bin was defined by a unique overlapping substitution segment from the CSSLs and used as a marker for QTL analysis. A linear ridge regression in the R package “ridge” (available online: http://www.r-project.org/) was applied for QTL analysis in the CSSL population [33]. In addition, *p* < 0.01 was set as the significance level for the presence of a putative QTL. The most significant bin was selected if several adjacent bins showed significant *p*-values. The phenotypic variance explained by each QTL (bin) was calculated using lmg in the R package named “relaimpo”.

A linkage map was constructed for the CSSL-derived F_2_ population with an additional 10 markers on the target region. Linkage analysis and QTL validation for seed dormancy were performed using the ICIMAPPING software (version 4.1, Chinese Academy of Agriculture Sciences, Beijing, China). The presence of a QTL was declared when an LOD score was larger than 3. The additive effect and the phenotypic variation explained by each QTL were estimated by ICIMAPPING.

### 4.5. Quantification of Endogenous ABA

About 100 mg of embryo from fresh seeds were extracted with 750 μL of methanol/water/acetic acid (80:19:1) and 10 ng/ ml of d6-ABA as internal standard, shaking for 16 h at 300 rpm in 4 °C. After centrifuging for 10 min at 13,000 rpm, the supernatant was transported to a new tube. The precipitate was re-extracted with 450 μL methanol/water/acetic acid (80:19:1) shaking for 4 h at 300 rpm in 4 °C. After centrifuging for 10 min at 13,000 rpm, the supernatant was combined with the previous one. The extracts were filtered with 22 μm filter membrane (Bizcomr, Nylon Syringe Filter, Guangzhou, China) and dried with N_2_. The residue was dissolved in 200μL of methanol and centrifuged for 15 min at 13,000 rpm under 4 °C. About 150 μL of supernatant were used for ultra-fast liquid chromatography (UFLC)/electrospray ionization/tandem mass spectrometry system (ESI/MS/MS) (Agilent 6520 QTOF, Hong Kong, China).

### 4.6. Exogenous ABA Treatment

A seed germination assay was performed as described above. For the ABA sensitivity assay, after-ripened seeds of NIL-NIP and NIL-ZS97 were treated with a series of ABA solutions (0, 1, 5, 10, 20, 50, and 100 μM). Dimethyl sulfoxide (DMSO) was used to dissolve ABA.

Subsequently, a 20 μM portion of ABA was chosen to treat after-ripened NIL-NIP and NIL-ZS97 to further measure the candidate gene expression. DMSO was used as control (CK). Embryos germinated for three days were used for RNA isolation.

### 4.7. RNA Isolation and qRT–PCR Analysis

RNA isolation: RNAPrep Pure Plant Plus Kit (TIANGEN, catalog number DP441, Beijing, China) was used to extract total RNA from embryos of germinated seeds, according to the manufacturer’s instructions. IScript cDNA Synthesis Kit (Bio-Rad, Hercules, CA, USA) was used for cDNA synthesis for quantitative real-time PCR (qRT-PCR), according to the manufacturer’s instructions. qRT-PCR was performed using a QuantStudio (TM) 6 Flex Real-Time PCR instrument (Applied Biosystems, Waltham, MA, USA) with iQ SYBR Green Supermix (Bio-Rad, Hercules, CA, USA). Three biological replicates were used for each sample. The data were normalized to the amplification of a rice ACTIN gene (LOC_Os03g50885). The mean value was then plotted with its standard error. Primers for real-time PCR are included in Table S2, Supplementary Materials.

## 5. Conclusions

Rice seed dormancy is an important agronomic trait that is crucial to the quality of rice. Currently, rice breeding programs have been focused on cultivars with a balance between pre-harvest sprouting and deep dormancy [2]. Cultivars with moderate dormancy levels have multiple advantages, including decreasing pre-harvest sprouting, improving the survival rate of direct seedling rice, increasing seedling growth uniformity, and further improving the storage quality of rice. The QTL *qDOM3.1* controlling seed dormancy identified in the present study will accelerate the breeding of new rice varieties with suitable seed dormancy. Besides *qDOM3.1*, many of the QTLs identified in the present study may also be useful in an agricultural context, providing new genes that can be used to improve crop performance under fluctuating environments.

## Figures and Tables

**Table 1:** Germination parameters of the two parents and the chromosomal segment substitution line (CSSL) population.

Index	Parents		CSSLs		
	NIP	ZS97	Mean ± SD	CV%	Min to Max
G_max_ (%)	37.7 ± 3.2**	100.0 ± 0.0	96.7 ± 7.03	7.26	36.4 to 100.0
G_3d_ (%)	0**	91.01 ± 6.7	92.3 ± 14.90	16.15	12.0 to 100.0
T_50_	-	36.8 ± 2.5	41.6 ± 9.66	23.22	24.4 to 91.0
U_8416_	47.9 ± 6.3**	11.2 ± 2.3	15.9 ± 11.21	70.57	4.14 to 75.4
AUC	19.2 ± 0.03**	130.3 ± 2.4	121.2 ± 15.24	12.57	45.1 to 141.9
GI	8.4 ± 0.5**	76.6 ± 2.9	23.5 ± 3.97	16.92	8.27 to 35.9

G_max_, maximum germination percentage of seven days of germination; G_3d_, germination percentage at three days; T_50_, time to reach 50% germination of the total number of germinated seeds; U_84-16_, germination uniformity, which is time interval between 84% and 16% of viable seed to germinate; AUC; area under the germination curve until 168 h; GI, germination index. SD, standard deviation; CV, coefficient variation; Min to Max, the minimum and maximum value in the CSSL population. Asterisks **, indicate significant difference between the parents at the level of 0.01.

**Table 2:** QTLs identified for six germination parameters in the NIP/ZS97 CSSL population using the single nucleotide polymorphism (SNP) bin markers.

Chr	Interval (Mb)	QTL	G_max_^a^			G_3d_			T_50_			U_8416_			AUC			GI			QTL/Gene^c^
			P^b^	PVE%	E	*p*	PVE%	E	*p*	PVE%	E	*p*	PVE%	E	*p*	PVE%	E	*p*	PVE%	E	
1	2.3–3.0	*qDOM1.1*	-^d^	-	-	-		-	4.4	0.9	1.59	3.0	1.7	1.46	-	-	-	-	-	-	
1	12.5–13.8	*qDOM1.2*	-	-	-	3.6	1.7	0.00	4.2	1.5	1.19	4.2	1.3	1.75	2.6	1.0	−0.37	3.1	1.5	−0.17	
1	19.5–20.3	*qDOM1.4*	-	-	-	3.3	1.8	0.00	3.4	1.7	1.49	4.0	2.1	1.85	2.7	1.2	−0.44	2.3	1.3	−0.18	
1	28.5–28.8	*qDOM1.5*	-	-	-	-	-	-	-	-	-	2.2	1.7	−2.03	2.5	1.2	−0.44	2.2	1.1	−0.09	
1	40.2–40.6	*qDOM1.6*	-	-	-	-	-	-	-	-	-	3.1	0.4	0.77	-	-	-	-	-	-	
2	6.7–6.9	*qDOM2.1*	-	-	-	-	-	-	3.5	3.9	0.67	3.0	2.0	0.69	-	-	-	2.2	1.6	−0.09	*qDOR-2*
2	12.1–23.1	*qDOM2.2*	-	-	-	-	-	-	2.7	1.0	−2.81	-	-	-	-	-	-	-	-	-	
2	23.9–24.9	*qDOM2.3*	2.2	2.1	−0.01	3.6	1.8	0.00	5.1	2.2	1.88	-	-	-	3.2	1.7	−0.53	3.0	1.8	−0.21	
3	0.2–2.1	*qDOM3.1*	3.8	4.9	−0.01	6.6	5.2	−0.01	4.9	5.1	2.88	9.2	8.3	4.46	6.6	6.4	−0.87	4.4	3.4	−0.30	
3	3.9–4.4	*qDOM3.2*	-	-	-	-	-	-	-	-	-	-	-	-	-	-	-	2.3	3.3	0.14	*qSD-3*
3	13.2–14.8	*qDOM3.3*	2.6	2.6	−0.01	4.9	3.1	0.00	4.1	3.5	1.86	7.2	5.5	2.78	4.5	3.7	−0.58	3.0	1.8	−0.19	*qSD-3-2*
3	14.8–16.9	*qDOM3.4*	2.3	2.6	−0.01	4.7	2.9	0.00	-	-	-	2.3	4.4	1.72	-	-	-	2.7	1.6	−0.18	
3	23.0–24.7	*qDOM3.5*	-	-	-	2.3	1.4	0.00	-	-	-	2.8	2.0	1.13	-	-	-	-	-	-	
3	36.2–36.4	*qDOM3.6*	-	-	-	-	-	-	-	-	-	2.3	1.1	−0.88	-	-	-	-	-	-	
4	5.5–6.0	*qDOM4.1*	-	-	-	-	-	-	3.1	1.4	−2.51	-	-	-	-	-	-	-	-	-	
4	34.6–4.9	*qDOM4.2*	-	-	-	5.4	3.0	0.00	2.5	3.6	0.90	-	-	-	3.1	1.3	−0.37	3.1	1.4	−0.15	
5	7.2–7.3	*qDOM5.1*	-	-	-	-	-	-	3.2	1.2	−1.13	-	-	-	-	-	-	-	-	-	
5	21.4–24.1	*qDOM5.2*	-	-	-	-	-	-	2.9	1.6	1.47	4.1	1.9	2.00	-	-	-	2.5	2.7	−0.19	
5	25.0–30.0	*qDOM5.3*	-	-	-	2.1	4.9	−0.01	4.9	3.0	3.50	3.2	2.3	3.00	2.6	5.6	−0.63	3.4	4.0	−0.33	
6	11.0–11.5	*qDOM6.1*	-	-	-	3.5	2.0	−0.01	-	-	-	-	-	-	2.8	1.5	−0.69	2.1	1.1	−0.26	
6	12.0–14.4	*qDOM6.2*	4.3	8.6	−0.02	8.2	7.4	−0.01	15.7	6.5	6.44	6.1	3.6	4.34	6.4	4.9	−1.47	4.8	3.3	−0.54	
7	0.0–0.5	*qDOM7.1*	-	-	-	4.0	2.4	−0.01	4.7	2.1	2.72	-	-	-	3.1	1.7	−0.72	2.3	1.2	−0.27	
7	2.4–2.8	*qDOM7.2*	-	-	-	-	-	-	2.7	1.1	−1.15	-	-	-	-	-	-	-	-	-	
7	4.9–5.2	*qDOM7.3*	-	-	-	3.9	2.2	0.00	3.2	1.7	1.10	2.4	3.1	0.96	3.0	1.5	−0.29	2.8	1.8	−0.12	
7	5.7–17.7	*qDOM7.4*	-	-	-	7.7	6.2	0.00	8.3	8.1	2.01	9.5	9.9	2.78	5.8	3.9	−0.55	5.6	4.7	−0.25	*qSD7-1/Rc*
7	17.7–18.5	*qDOM7.5*	10.5	9.6	−0.01	5.4	6.3	0.00	-	-	-	-	-	-	6.7	8.3	−0.54	5.9	6.7	−0.21	
7	19.2–19.5	*qDOM7.6*	9.3	7.2	−0.01	5.9	7.3	0.00	-	-	-	-	-	-	6.4	7.5	−0.61	4.5	3.4	−0.22	*qSD.7*
7	22.6–24.4	*qDOM7.5*	-	-	-	-	-	-	4.0	1.4	−1.36	-	-	-	-	-	-	-	-	-	*Sdr4*
8	8.1–19.1	*qDOM8.1*	-	-	-	2.4	4.6	−0.01	5.6	5.0	5.04	-	-	-	-	-	-	-	-	-	
9	13.8–15.0	*qDOM9.1*	-	-	-	-	-	-	3.4	3.7	1.60	2.2	1.7	1.72	-	-	-	-	-	-	*qDOR-9-1*
9	21.5–22.6	*qDOM9.2*	-	-	-	-	-	-	-	-	-	2.1	1.2	0.77	-	-	-	-	-	-	*qDOR-9-2*
10	0.1–1.4	*qDOM10.1*	-	-	-	-	-	-	-	-	-	3.8	3.6	1.38	-	-	-	-	-	-	
10	6.4–10.3	*qDOM10.2*	2.1	1.3	0.00	6.0	4.9	0.00	3.8	5.9	1.22	6.4	7.3	1.78	5.4	5.4	−0.43	4.7	4.7	−0.17	
10	13.2–20.3	*qDOM10.3*	8.2	11.6	−0.02	10.6	11.5	−0.01	15.7	10.4	5.44	15.7	12.5	6.87	10.7	12.9	−1.33	6.7	5.8	−0.44	*OsFbx352*
11	6.5–7.2	*qDOM11.1*	-	-	-	-	-	-	2.7	0.4	−1.00	4.5	0.7	1.37	-	-	-	-	-	-	
12	1.1–3.0	*qDOM12.1*	-	-	-	-	-	-	5.2	3.6	9.13	10.8	8.8	14.90	-	-	-	-	-	-	*qSD12*

a. G_max_: maximum germination percentage of seven days of germination, G_3d_: germination percentage at three days, T_50_: germination speed, which is time to reach 50% germination of the total number of germinated seeds, U_8416_: germination uniformity, which is time interval between 84% and 16% of viable seed to germinate, AUC: area under the germination curve, GI: germination index; b. When two or more consecutive bins were significant, the lowest *p*-value was selected and displayed as –log10(P). PVE presents the phenotypic variance explained for a given QTL. E indicates estimated additive effect by a given QTL. For G_max_, G_3d_, AUC, and GI, the positive E value represents the NIP alleles that increased the effect, and for T_50_ and U_8416_ the positive E value represents the ZS97 alleles that increased the effect; c. The genes near 500 kb of the most significantly associated SNP; d. - Indicates no QTLs detected.

**Table 3:** Confirmation of *qDOM3.1* loci by NQ96.

	G_max_%	G_3d_%	T_50_ (h)	U_8416_ (h)	AUC	GI
ZS97	100 ± 0	91.01 ± 6.7	36.76 ± 2.48	11.22 ± 2.26	130.29 ± 2.37	76.63 ± 2.97
NQ96	57 ± 7.07**	30 ± 8.49**	97.03 ± 9.79**	32.11 ± 11.82*	63.97 ± 10.13*	35.48 ± 6.36**
NIP	37.71 ± 3.24	0	-	47.9 ± 6.25	19.22 ± 0.03	8.42 ± 0.46

Germination behaviors that are significantly different from that of ZS97 are indicated by asterisks (* *p* < 0.05, ** *p* < 0.01). - no data available.

## Supplementary Materials

Supplementary materials can be found at https://www.mdpi.com/1422-0067/21/4/1344/s1.

## Abbreviations

AUC	Area Under the Germination Curve
CSSL	Chromosomal Segment Substitution Line
GI	Germination Index
G_max_	Maximum Germination Percentage of Seven Days of Germination
G_3d_	Germination Percentage at Three Days
NIL	Near-Isogenic Line
T_50_	Germination Speed, which is the time to reach 50% germination of the total number of germinated seeds
U_8416_	Germination Uniformity, which is the time interval between 84% and 16% of viable seed to germinate
