# Supplementary material for: Genetic Dissection of Seed Dormancy using Chromosome Segment Substitution Lines in Rice (Oryza sativa L.)
Source: Int J Mol Sci. 2020 Feb 17;21(4):1344. doi: 10.3390/ijms21041344 (PMC7072991; doi:10.3390/ijms21041344)
Supplement: Supplementary file 1 [file ijms-21-01344-s001.pdf]

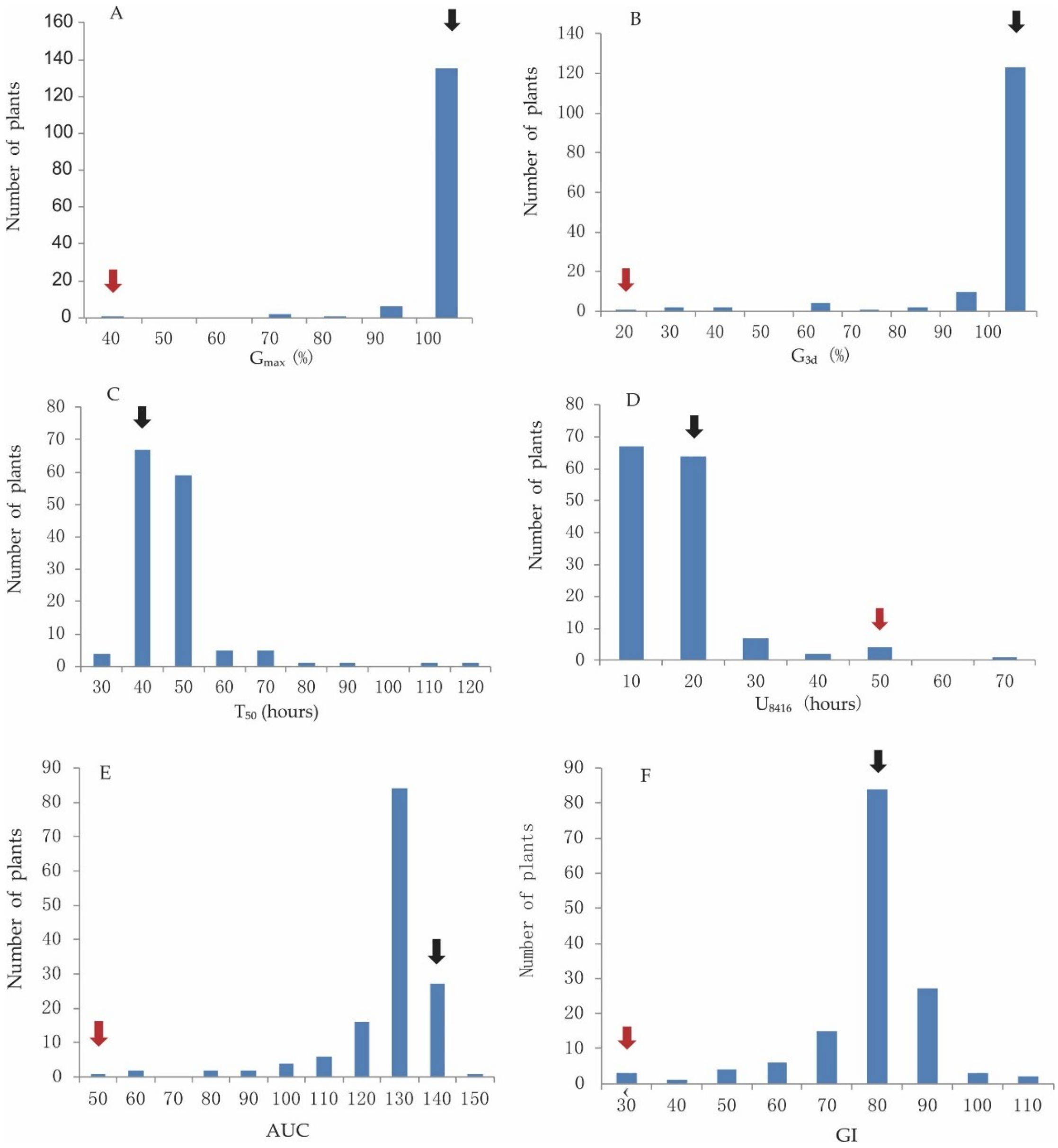

Figure S1. Frequency distribution of (A) G_{max}, maximum germination percentage of seven days germination; (B) G_{3d}, germination percentage at three days; (C) T_{50}, time to reach 50% germination of the total number of germinated seeds; (D) U_{8416}, germination uniformity, which is time interval between 84% and 16% of viable seed to germinate; (E) AUC; area under the germination curve until 168 h; (F) GI, germination index. Black arrow indicates ZS97 performance and red arrow indicates NIP performance.

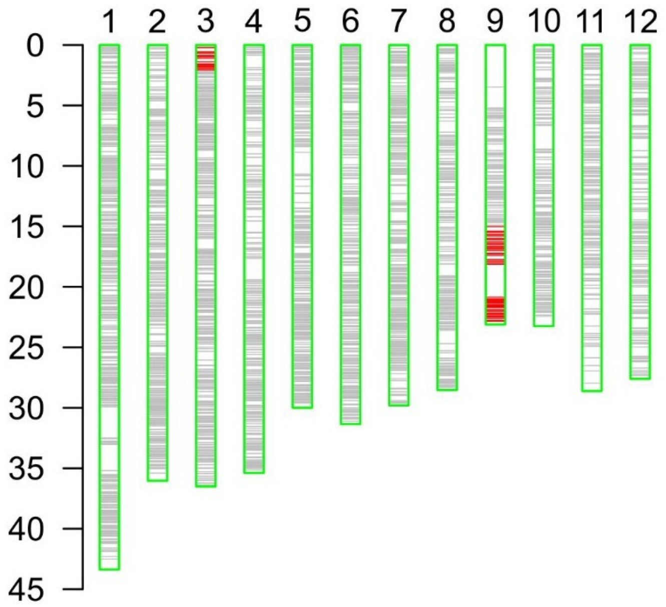

Figure S2. Graphic representation of the genotype of NQ96 showing several introduced segments, of which the segment on chromosome 3 harbors the target QTL.

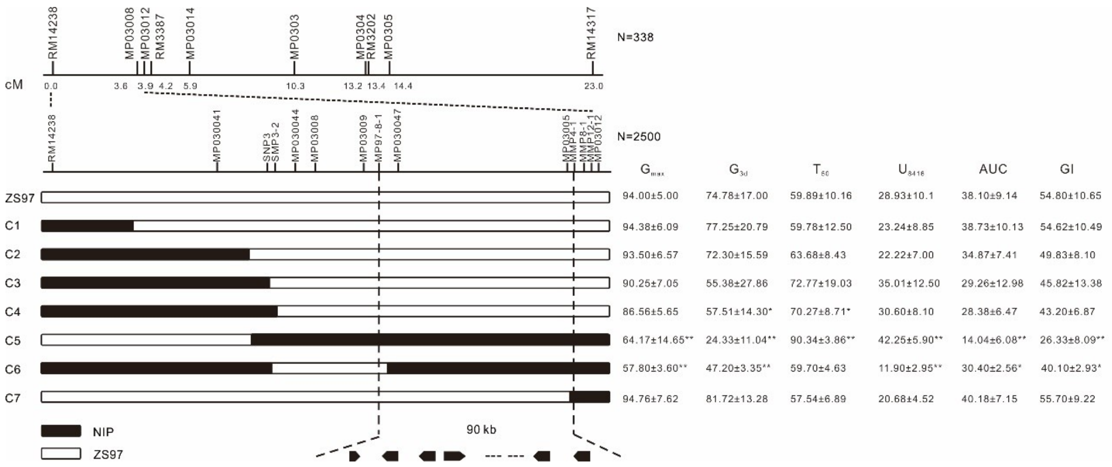

Figure S3. Fine mapping of *qDOM3.1*. The QTL narrowed down to the region flanked by MP97-8-1 and MMP4-1 on the upper end of chromosome 3. Some important recombinant plants derived from a large F_2 groups generated by selfing a single individual heterozygous at the *qDOM3.1* region and divided into 7 groups based on their genotypes. G_{max} (mean ± sd) (%) at 168 hours, G_{3d}, T_{50}, U_{8416}, AUC and GI was given on the right for each genotype. The phenotypes of each recombinant individual were evaluated by germination experiments. * and ** indicate significant difference at $p < 0.05$ and $p < 0.01$ by Dunnett's test against ZS97, respectively.

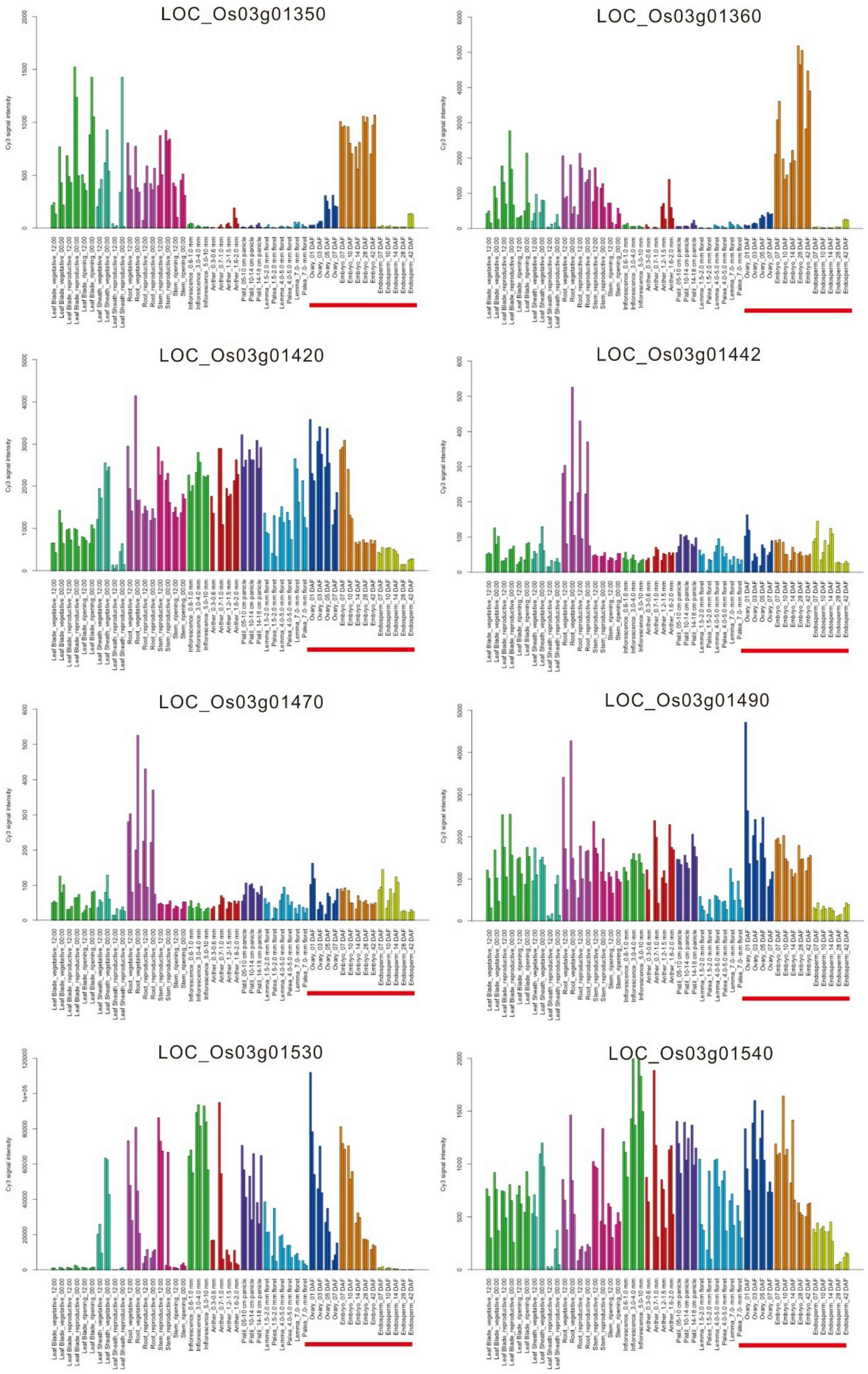

Figure S4. The eight candidate gene expression profile (http://ricexpro.dna.affrc.go.jp/). The red bar in each figure indicate the expression in seeds.

Table S1. Summary information on AS-PCR, SSR and Indel markers for *qDOM3.1* validation and fine-mapping.

Marker	Type	Forward primer	Reverse primer	CHR
SNP3	AS-PCR	CGGGAGCGGCTGTGCGGTAG	AGCAGCTTCTTGAAGCGGTAGGTGGT	3
SNP3	AS-PCR	CGGGAGCGGCTGTGCGGTAT	AGCAGCTTCTTGAAGCGGTAGGTGGT	3
SMP-3-2	SNP	AGCCTAGTATGTGGCCCTGAA	CATTCCACGCCATTAAACA	3
MMP97-8-1	SNP	ATGATTGATACGGTTCTCG	ACTTATTCCTGGGTGCTAC	3
MMP-4-1	SNP	CAGAAGCAAGGGTTACACG	CAGGAAATCATACCGAAGA	3
MMP-8-1	SNP	TTAAGGGTCAAATGTCTGT	ACATCTTGTCCGTGGTCTG	3
MMP-12-1	SNP	TCTACCTCCCGCTGCTAAT	TCCGTCGCTTCATTCTAAC	3
MP0303	Indel	GGGGAAAACGAAGAATAAGT	GCTTGATTAATGGCAAGAAC	3
MP0304	Indel	AGGAACACATACGAATGGAG	AGAGCATCATGCAGGTCC	3
MP0305	Indel	GGTTATGATTCGTTGGAAAA	AAACAAATACTCCCTCTCAAAA	3
MP03008	Indel	TTTGGTCTGTATCCCATTTG	TCATAGGTCACGATCTTGCT	3
MP03009	Indel	GCAGCAACAAAAGAGTAACG	TCATGTGTGGGGTAATCTGT	3
MP03012	Indel	GCACTGTGAACACCCTTACA	CCTGAAACGGAGGGATTA	3
MP03014	Indel	TTCAACACAGAAGTTCACCA	CAATAGCTACGTCCTCCTGA	3
MP030041	Indel	ACCACTAATACTAGCAGCAGG	TAGAGGAGGGGTACTTTCA	3
MP030044	Indel	TACAAATAGAAAGCAAAGGAG	TCATGTTAAGTATCCCACAAA	3
MP030047	Indel	CTAGTCCGTGATATGGAAGG	CCACCCAGTTGTTGATTATT	3
MP030050	Indel	CACTGATGAACAGCCATTCT	TGATGATAGGCACAATACCC	3
RM3202	SSR	TCATCATCATCAGTCCAGCATCG	GCGGCGATTGAATTGTTTCTTAGG	3
RM3387	SSR	GTGTAGCAGCAGCTGGACAACC	GTGCGAGATCGAGGCAAATACG	3
RM14317	SSR	TGAATTCTTGCACATGGTCAGC	TGGGAGGTTTGCTAGGGTAATCC	3
RM14238	SSR	CCGTCTCTCTGTTGTTGTTCTGC	TGGTTCGCCAACAATTAAGACG	3

Table S2. Primers for Real-Time PCR.

Gene	Forward primer	Reverse primer
LOC_Os03g01350	TTGATTGCAGCCTTTGGTGTTG	TCGTTCCCTGGTTGAATCCTTA
LOC_Os03g01360	TTCTGCACGGCAGTGGAGTC	TGAAACCTATCGCCTGGGAG
LOC_Os03g01420	CTCTAACATCTGCATATCCCACA	GATTCCCATTACCGACTTCC
LOC_Os03g01442	GGATGCGAGCGTATGGTGTC	TGCGTCAGAAAAGTTGGAAG
LOC_Os03g01470	GATGGTAGGGGCGAGGGG	GCCACCCTGACGCCAATC
LOC_Os03g01490	AGGGTTGAGTATGTGCCTGAG	GAGGTTAGAAGCAGCCAAGTAG
LOC_Os03g01530	GGGCCAAGGGACACTACACC	CCAAGCGAGTGGCAAACCTG
LOC_Os03g01540	GCCTCCAGCCCCACCTTCTC	CACCCTCCCATTTGACTTTG
OsActin	GAATGCTAAGCCAAGAGGAG	AATCACAAGTGAGAACCACAG
